# Protein “purity,” proteoforms, and the albuminome: critical observations on proteome and systems complexity

**DOI:** 10.3389/fcell.2024.1504098

**Published:** 2024-12-10

**Authors:** Breyer Woodland, Jens R. Coorssen, Matthew P. Padula

**Affiliations:** ^1^ Proteomics, Lipidomics and Metabolomics Core Facility, School of Life Sciences, Faculty of Science, University of Technology Sydney, Ultimo, NSW, Australia; ^2^ Department of Biological Sciences, Faculty of Mathematics and Science, Brock University, St. Catharines, ON, Canada; ^3^ Institute for Globally Distributed Open Research and Education (IGDORE), St. Catharines, ON, Canada

**Keywords:** biologics, biomarkers, bovine serum albumin, integrative top-down proteomics, proteoforms, tandem mass spectrometry, therapeutics, two-dimensional gel electrophoresis

## Abstract

**Introduction:**

The identification of effective, selective biomarkers and therapeutics is dependent on truly deep, comprehensive analysis of proteomes at the proteoform level.

**Methods:**

Bovine serum albumin (BSA) isolated by two different protocols, cold ethanol fractionation and heat shock fractionation, was resolved and identified using Integrative Top-down Proteomics, the tight coupling of two-dimensional gel electrophoresis (2DE) with liquid chromatography and tandem mass spectrometry (LC-MS/MS).

**Results and discussion:**

Numerous proteoforms were identified in both “purified” samples, across a broad range of isoelectric points and molecular weights. The data highlight several concerns regarding proteome analyses using currently popular analytical approaches and what it means to (i) purify a “protein” if the isolate consists of a wide variety of proteoforms and/or co-purifying species; and (ii) use these preparations as analytical standards or therapeutics. Failure to widely recognize and accept proteome complexity has likely delayed the identification of effective biomarkers and new, more selective drug targets. iTDP is the most logical available analytical technique to effectively provide the necessary critical depth and breadth for complex proteome analyses. Routine analyses at the level of proteoforms will provide the much-needed data for the development and validation of selective biomarkers and drugs, including biologics.

## 1 Introduction

Biomarkers and therapeutics are meant to be highly selective agents. Regrettably, this has, over decades, not often proven to be the case. This problem is reflected in the low number of clinically effective molecular biomarkers and in both the high failure rates during drug development and the off-target effects of most, even “cutting-edge,” therapeutics. Much of this is linked to the lack of truly deep, comprehensive analysis of proteomes and the somewhat short-sighted and “easier” focus on canonical protein sequences (Carbonara et al., 2021; Coorssen, 2023a; Coorssen, 2023b; Coorssen and Padula, 2024). This again raises the very obvious questions: what does it actually mean to measure changes in the abundance of a “protein” and what does it mean to isolate or purify a “protein”?

Protein species or variants – including but not limited to mutations, alternate splicing, mRNA processing, and any posttranslational modifications (PTM) to an amino acid sequence - broadly termed proteoforms, are the drivers of physiology, explaining how a relatively limited genome can account for the complexity of a system and define functions from the level of molecular interactions to individual, whole organism phenotypes. Thus, proteoform analysis is the logical basis for the identification of more appropriately selective biomarkers, therapeutic targets, and drugs, including biologics (Coorssen and Yergey, 2015; Xu et al., 2018; Carbonara et al., 2021; Kjer-Hansen et al., 2024). However, the most common current analytical approaches—shotgun or bottom-up proteomics (BUP) and mass spectrometry-intensive top-down proteomics (MSi-TDP) — both fail to fully and effectively address this analytical dilemma, either inferring the presence of intact canonical sequences, being ineffective for identifying proteoforms, and/or being unable to provide comprehensive proteoform analysis across the whole proteome (Coorssen, 2023b; Coorssen and Padula, 2024). Currently, only high-resolution, quantitative integrated/integrative TDP (iTDP; 2D gel electrophoresis (2DE) coupled with liquid chromatography and tandem mass spectrometry (LC-MS/MS)) can effectively provide the depth and breadth of proteome analysis necessary to effectively assess proteoforms (Coorssen and Yergey, 2015; Carbonara et al., 2021; Coorssen and Padula, 2024). To highlight the complexity of the issue, here we focus on serum albumin which is widely used as a biomarker (e.g., microalbumin) and therapeutic, while also being linked to the development of diabetes, (cardio)metabolic syndrome, and other serious disorders (Taverna et al., 2013; Bhat et al., 2017; Jun et al., 2017; Sharma et al., 2023; Hao et al., 2024). Additionally, with the description of the so-called albuminome (ostensibly an interactome), this potential sub-proteome is also of interest (Zhou et al., 2004; Gundry et al., 2007; Scumaci et al., 2011; Liu et al., 2017).

Although albumin species have been known for some time, with the exception of a few studies, most notably identifying glycosylated or glycated variants, relatively little attention has been paid to albumin proteoforms more broadly (Coussons et al., 1997; Kawakami et al., 2006; Rondeau and Bourdon, 2011; Marie et al., 2013; Leblanc et al., 2018; Smith et al., 2023). This is also a prime example of the self-imposed limitations of most current studies in proteomics, and how they fail to appreciate the need for routine, *comprehensive* analyses at the level of proteoforms. In effect, like other “proteins,” albumin is most generally thought of as a single molecular entity and treated as such; this is particularly true in terms of peptide, drug, and small molecule binding studies. Despite the current popularity of BUP analyses there is a critical and increasingly obvious need to understand molecular diversity in terms of proteoforms. This is essential to the specificity needed to develop better, more refined and optimized clinical products/biologics, biomarkers, and therapeutics.

Here, for ease of access to what are considered highly purified samples, bovine serum albumin (BSA) is used as an analytical surrogate for human serum albumin (HSA), noting the high sequence and structural similarity of the two primary open reading frame (ORF) products (i.e., canonical sequences). Analysis of BSA isolated by two different protocols, cold ethanol fractionation (CEF) and heat shock fractionation (HSF), both of which are also routinely used to purify HSA, reveals a substantial spectrum of variants, most of which prove to be proteoforms rather than unrelated, co-purifying species. As the study deals with molecular separations and analyses from the critical perspective of comprehensive iTDP and systems biology, rather than for commercial purposes, here we prefer the term co-purifying as opposed to contaminating species as it better reflects the inherent issues of similarities in physicochemical properties and what must therefore be considered more carefully in (i) designing experiments to identify proteoforms and genuine interacting species; and (ii) genuinely purifying select active species of interest in specific conditions. Using purified BSA, a relatively “simple system,” we highlight the complexity of proteome analyses and emphasize the pressing need for deep, comprehensive analysis of proteoforms to identify highly selective biomarkers and therapeutic targets, and ensure the purity of biologics.

## 2 Materials and methods

All consumables were of electrophoresis-grade or higher. Electrophoresis equipment, ReadyStrip™ IPG Strips (7 cm, pH 3–10 non-linear), BioLyte^®^ 3/10 Ampholytes, 40% acrylamide/bis-acrylamide (37.5:1) solution, Coomassie Brilliant Blue (CBB) G-250 powder, acrylamide powder, CHAPS, and Precision Plus Protein™ Unstained Standards (10–250 kDa) were obtained from Bio-Rad Laboratories. Lyophilized Bovine Serum Albumin (BSA) purified by cold-ethanol fractionation (product no. A7517, lot no. SLCM2607) or heat-shock fractionation (product no. A8022, lot no. SLBC0344V), urea, thiourea, sodium n-dodecyl sulfate (SDS), glycerol, tributylphosphine (TBP), dithiothreitol (DTT), citric acid, trifluoroacetic acid (TFA), acetonitrile (ACN), tris hydrochloride, acetic acid, ammonium bicarbonate (AMBIC) and Roche cOmplete™ Mini EDTA-free Protease Inhibitor (PI) Cocktail tablets were purchased from Sigma-Aldrich. Mass Spectrometry Grade Trypsin Gold was purchased from Promega. Milli-Q water was used throughout.

### 2.1 Sample preparation

The lyophilized BSA samples were solubilized in Milli-Q water as previously described (Gauci et al., 2013; Noaman et al., 2017). Protein concentrations were measured using the Thermo Scientific™ NanoDrop™ One Microvolume UV-Vis Spectrophotometer. Gel-based purity analysis was carried out as previously described (Noaman et al., 2017).

### 2.2 2DE: Isoelectric focusing (IEF) and SDS-PAGE

For each BSA sample, three 2DE replicates were resolved. Prior to passive rehydration of microneedled IPG strips, 10 μg of BSA in 8 M urea, 2 M thiourea, 4% *(w*/*v)* CHAPS, and 1X PI was reduced with 100 mM DTT +5 mM TBP at 25°C for 1 h, followed by alkylation with 230 mM of acrylamide for 1 h (Carbonara and Coorssen, 2023; Woodland et al., 2023). Rehydrated IPG strips were focused at 17°C, as previously described (Butt and Coorssen, 2005; Butt et al., 2007). Following IEF, IPG strips were equilibrated with 6 M urea, 20% (*w*/*v*) glycerol, 2% (*w*/*v*) SDS, and 375 mM Tris [pH 8.8], and incubated with 130 mM DTT for 10 min, and then with 350 mM acrylamide for 10 min. SDS-PAGE (12%T mini-gel format) was carried out as previously described (Noaman et al., 2017) and all gels were then fixed in 1 M citric acid in 5% (*v*/*v*) acetic acid for 1 h at RT with gentle rocking (Carbonara and Coorssen, 2020). Gels were then washed in Milli-Q water (3 × 20 min washes). To identify the sub-proteomes associated with phosphorylation and glycosylation, one replicate of each resolved BSA sample (i.e., HSF and CEF) was stained with Invitrogen™ Pro-Q™ Diamond (phosphoproteoforms) and one with Pierce™ Glycoprotein Staining Kit (glycoproteoforms), respectively, according to manufacturer’s protocols. Stained gels were imaged using the GE Healthcare Typhoon FLA 9500 Biomolecular Imager for phosphoproteoforms (532/575 nm excitation/emission, 50 μm pixel size, PMT gain set to 600 V) and Amersham Imager 600 for glycoproteoform detection (Colorimetric capture, white light epi*-*illumination). Following these PTM stains, gels were washed in Milli-Q water (3 × 20 min washes), then stained with a colloidal Coomassie Brilliant Blue (cCBB) solution for total proteoform detection, as previously described (Gauci et al., 2013; Noaman et al., 2017). The third replicate gel of each sample was stained only using cCBB for total proteoform detection. cCBB-stained gels were destained with 0.5 M NaCl (5 × 15 min washes) prior to imaging by near-infrared fluorescence detection (nIRFD) using a GE Healthcare Typhoon FLA 9000 Biomolecular Imager with 685 nm excitation laser, 713–726 nm emission filter (BPFR700, GE Healthcare), 50 μm pixel size, and PMT gain set to 600 V (Butt and Coorssen, 2013; Noaman et al., 2017; Carbonara et al., 2023).

### 2.3 In-gel digestion and peptide clean up.

Coomassie-stained spots from one gel replicate were manually excised and destained by washing twice in destain solution (50% *(v/v)* ACN/50 mM AMBIC [pH 9]) for 10 min with vortexing. In addition, a series of 5 gel blanks were excised from apparently proteoform-free regions of each gel. After the destain solution was removed, the gel pieces were dehydrated with 100% *(v/v)* ACN for 10 min. Gel pieces were rehydrated with 25 μL 100 mM AMBIC [pH 9] containing 3 ng/μL trypsin at RT for 30 min. An additional 25 μL of 100 mM AMBIC [pH 9] was added and gel pieces were incubated overnight at 4°C (Wright et al., 2014b). Peptides were recovered using SDB-RPS-based stage tips as previously described, with some modifications (Rappsilber et al., 2007). In-gel digested spots were sonicated using a bath sonicator for 10 min, followed by the addition of 150 μL SPE Load Buffer (90% *(v/v)* ACN, 1% *(v/v)* TFA) and sonicated for an additional 10 min. The digest in SPE Load Buffer was added to the top of a SDB-RPS-based STAGE tip and the liquid was centrifuged through at 5,000 rpm for 2 min, or until all the liquid passed through. Bound peptides in the STAGE tip were washed once with 100 μL SPE Load Buffer by centrifuging at 5,000 rpm for 2 min, or until all the liquid passed through. Bound peptides were washed again with 100 μL SPE Wash Buffer (10% *(v/v)* ACN, 0.1% *(v/v)* TFA) by centrifuging at 5,000 rpm for 2 min, or until all the liquid passed through, to remove any contaminants and salts. Peptides were eluted directly into MS injection vial inserts with 50 μL SPE Elution Buffer (80% *(v/v)* ACN, 71 mM ammonium bicarbonate). Peptides in SPE elution buffer were evaporated to dryness using the Savant™ DNA 120 SpeedVac™ Concentrator. Dry peptides were reconstituted in 5 μL of MS loading solvent (2% *(v/v)* ACN, 0.2% *(v/v)* TFA) and stored at 4°C until analysed by LC-MS/MS.

### 2.4 LC-MS/MS

The sequence of gel spot digests was randomized prior to LC-MS/MS and “cleans” (injections of 1:1:1:1 water/ACN/methanol/isopropanol with 0.2% formic acid) were utilized after high abundance spots to ensure there was no peptide carry-over between sample injections. Using an Acquity M-class nanoLC system (Waters, United States), 5 µL of the sample was loaded at 15 μL/min for 3 min onto a nanoEase Symmetry C18 trapping column (180 μm × 20 mm) before being washed onto a PicoFrit column (75 µm ID × 100 mm; New Objective, Woburn, MA) packed with SP-120–1.7-ODS-BIO resin (1.7 µm, Osaka Soda Co., Japan) heated to 45°C. Peptides were eluted from the column and into the source of a Q Exactive Plus mass spectrometer (Thermo Scientific) using the following program: 5%–30% MS buffer B (98% ACN +0.2% Formic Acid) over 15 min, 30%–80% MS buffer B over 3 min, 80% MS buffer B for 2 min, 80%–5% for 3 min. The eluting peptides were ionized at 2400 V. A Data Dependent MS/MS (dd-MS2) experiment was performed, with a survey scan of 350–1,500 Da performed at 70,000 resolution for peptides of charge state 2+ or higher with an AGC target of 3e6 and maximum injection time of 50 m. The top 12 peptides were selected and fragmented in the HCD cell using an isolation window of 1.4 m/z, an AGC target of 1e5 and maximum injection time of 100 m. Fragments were scanned in the Orbitrap analyser at 17,500 resolution and the product ion fragment masses measured over a mass range of 120–2000 Da. The mass of the precursor peptide was then excluded for 30 s.

### 2.5 Data analysis

The MS/MS data files were searched using Peaks Studio 11 (Bioinformatic Solutions Inc.) against the UniProt *Bos taurus* (Bovine) reference proteome (downloaded 8 April 2024) and a database of common contaminants with the following parameters: Precursor mass error tolerance: 10.00 ppm. Fragment mass error tolerance: 0.02 Da. Enzyme: Trypsin. Maximum missed cleavages: 2. Digest-mode: Semi-specific. Peptide length range: 6–45. Fixed modifications: none. Variable modifications: Propionamide, Oxidized Methionine, and Deamidated Asparagine and Glutamine. Maximum variable PTM per peptide: 4. Peptide spectrum match (PSM) false discovery rate (FDR): 1.0%. Protein Group FDR: 1.0%. PEAKS PTM algorithm was used to identify PTM from the Unimod database for high-confident *de novo* scoring peptides that were not assigned in database searching. To confidently determine modification sites, the modified peptide must have an Ascore, the localization score assigned to modifications on the peptide, greater than or equal to 20 (p-value < 0.01) and an ion intensity ≥2 percent.

Following database searching, proteoform identification was determined based on the total number of peptides, sequence coverage, and protein confidence score. To confidently identify a proteoform, a minimum of three peptides was required (Coorssen and Yergey, 2015). Proteoforms identified by less than 3 peptides are reported in supplementary data (Supplementary Tables S1, S2). Unique peptides are defined as peptides that mapped to a single canonical protein on the day the database was interrogated (8 April 2024). PTM induced by sample preparation–propionamide from alkylation with acrylamide, oxidation of methionine, or deamidation of asparagine and glutamate are not specified by amino acid residue.

## 3 Results

**FIGURE 1:**
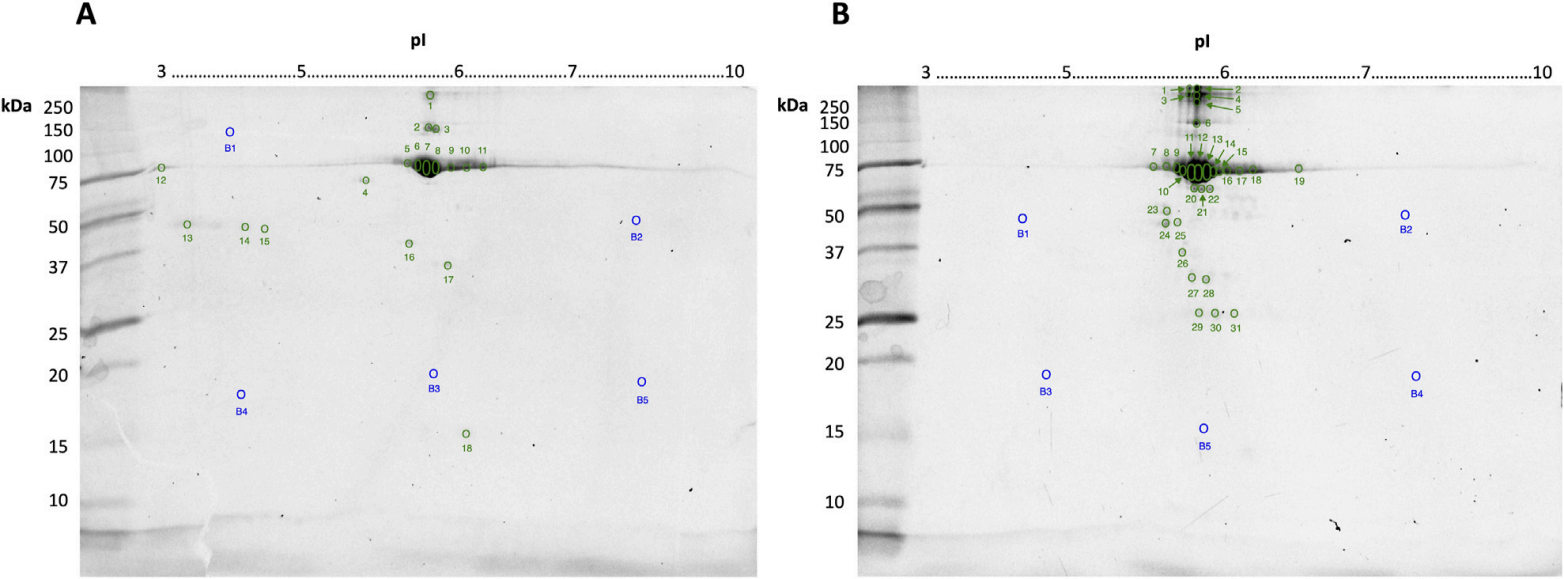
Representative gel images of 10 μg bovine serum albumin (BSA) purified by CEF **(A)** or HSF **(B)** resolved in the first dimension by IEF on 7 cm 3–10 non-linear IPG strips, and in the second dimension by SDS-PAGE using a 12.5% resolving gel and stained by cCBB. Numbers indicate the spots manually excised, digested and identified using LC-MS/MS, as listed in Tables 1, 2, respectively. Spot numbers (blue) with the letter “B” indicate gel blanks–apparently “proteoform-free” regions of the gel, as indicated by fluorescence imaging.

Commercially “purified” BSA stocks obtained by CEF or HSF were resolved by 2DE and stained for phosphoproteoforms, glycoproteoforms, and for total proteoform detection by cCBB. All replicate gel images, including those stained for phospho- and glycoproteoforms, are available in supplementary data (Supplementary Figures S1–S3). Total protein load per gel was 10 μg to enable adequate detection of lower abundance proteoforms by cCBB staining and aid in manual spot excision while ensuring the high abundance spot(s) at ∼70 kDa/pH 6 were not so over-saturated as to cause overlap and thus undue distortion of resolved adjacent spots. Following high-resolution imaging, all spots visible by eye were excised from the gels stained for total proteoforms by cCBB; total fluorescence signal was not significantly different between replicates (Figure 1).

Despite the lack of visible spots on the gels stained for phospho- and glycoproteoforms (Supplementary Figures S2, S3), MS analyses did extend to PTM (i.e., phosphorylated RPCFSALTPDETYVPK detected in spot B12, Supplementary Figure S4). Therefore, here, the lack of in-gel PTM detection is likely a result of the lower sensitivity of the stains available–notably the colorimetric glycoproteoform stain–and the lower 10 μg total protein loads used (as opposed to 100 μg loads usually used for total proteome analyses in the mini-gel format) (Wright et al., 2014a).

Twenty-eight spots and 42 spots (including gel blanks) were excised from the 2DE gels of BSA purified by CEF and HSF, respectively, in-gel digested with trypsin, and identified by LC-MS/MS. In both gels, albumin was identified in the majority of spots (Tables 1, 2). Distribution of species across the gel, with clear differences between observed and theoretical MW and pI, indicated the presence of multiple albumin proteoforms. Co-purifying species, or rather peptides thereof, were also identified in several spots and included vitamin D binding protein, bovine cytoskeletal keratins, and alpha-1-acid glycoprotein. No ORF products were identified in gel blanks with the exception of spot B5 (Figure 1B) from the HSF sample, in which albumin was identified, indicating that still more albumin proteoforms are present and though capable of being resolved by 2DE, were below the limit of detection at the low total protein load used. Spots with blank proteoform identification entries indicate that no non-contaminant peptides were identified (e.g., spots A16 and A18; Table 1; Figure1A).

**TABLE 1:** Spots excised from CEF BSA resolved by 2DE and the proteoforms identified by LC-MS/MS with ≥3 identified peptides**.** All identified proteoforms were from the Bos taurus species. MW, molecular weight; pI, isoelectric point; PTM, post translational modification. Propionamide (C), oxidation (M), or deamidation (NQ) are not specified by amino acid residue as they are likely artifacts of sample preparation.

Spot ID	Observed MW (kDa)/pI	Theoretical MW (kDa)/pI	Protein identified	Accession number	Gene	Protein confidence score (-10LgP)	Sequence coverage (%)	Number of peptides/Unique peptides	Area of sample	PTM
A1	>250/5.9	69.3/5.8	Albumin	P02769	ALB	357.27	72.65	68/68	2.02E+07	Propionamide (C), Oxidation (M), Deamidation (NQ)
A2	105.8/5.9	69.3/5.8	Albumin	P02769	ALB	266.56	27.84	19/19	7.71E+06	Propionamide (C), Oxidation (M)
A3	103.9/6	69.3/5.8	Albumin	P02769	ALB	401.42	78.42	89/89	7.71E+07	Propionamide (C), Oxidation (M), Deamidation (NQ), Glycidamide Adduct (L483), Carboxyethyl (K100, K437)
A4	53.8/5.5	69.3/5.8	Albumin	P02769	ALB	254.91	36.08	18/18	2.48E+06	Propionamide (C), Oxidation (M), Deamidation (NQ)
		53.3/5.4	Vitamin D-binding protein	Q3MHN5	GC	195.26	18.78	8/8	1.68E+06	Propionamide (C)
A5	64.2/5.8	69.3/5.8	Albumin	P02769	ALB	556.65	85.17	206/206	4.65E+08	Propionamide (C), Oxidation (M), Deamidation (NQ), Glycidamide Adduct (C125, C223, L483), Formylation (H402), Carboxyethyl (C437, K548)
A6	64.2/5.9	69.3/5.8	Albumin	P02769	ALB	713.51	92.09	352/350	5.95E+09	Propionamide (C), Oxidation (M), Deamidation (NQ), Glycidamide Adduct (C125, L529), Dehydration (T550), Acetylation (S296, D387), Formylation (R508)
A7	64.2/5.9	69.3/5.8	Albumin	P02769	ALB	730.13	92.09	427/421	9.62E+09	Propionamide (C), Oxidation (M), Deamidation (NQ), Methylation (K318), Carboxylation (K548), Acetylation (D387), Glycidamide Adduct (C223, L529), Formylation (Q413) Carboxyethyl (K100)
A8	64.2/6	69.3/5.8	Albumin	P02769	ALB	702.09	92.75	369/365	4.71E+09	Propionamide (C), Oxidation (M), Deamidation (NQ), Glycidamide Adduct (C125, C223), Acetylation (S296, D38), Dehydration (T550), Carboxyethyl (K100), 2-amino-3-oxobutanoic acid (Y161), Dehydration (T550), Amidation (F43)
A9	64.2/6	69.3/5.8	Albumin	P02769	ALB	564.59	87.64	206/205	8.22E+08	Propionamide (C), Oxidation (M), Deamidation (NQ), Glycamide Adduct (Q118, C125, C223, Dihydroxy (Y161), Acetylation (S296), Carboxyethyl (K548), 2-amino-3-oxobutanoic acid (Y161)
A10	64.2/6.1	69.3/5.8	Albumin	P02769	ALB	546.24	82.54	166/164	3.41E+08	Propionamide (C), Oxidation (M), Deamidation (NQ), Glycamide Adduct (C125), Acetylation (Q118), Formylation (H402), 2-amino-3-oxobutanoic acid (Y161)
		54.8/5.1	Keratin, type I cytoskeletal 10	P06394	KRT10	154.87	6.46	3/1	1.33E+04	
A11	64.2/6.2	69.3/5.8	Albumin	P02769	ALB	302.51	49.59	44/44	1.39E+07	Propionamide (C), Oxidation (M), Deamidation (NQ)
A12	64.2/3.1	69.3/5.8	Albumin	P02769	ALB	66.44	11.53	9/9	4.29E+05	Propionamide (C)
A13	39.6/3.3	69.3/5.8	Albumin	P02769	ALB	389.12	75.78	57/57	1.51E+07	Propionamide (C), Oxidation (M)
A14	39.2/4.2	69.3/5.8	Albumin	P02769	ALB	346.58	75.29	48/47	1.68E+07	Propionamide (C), Oxidation (M)
		23.1/5.6	Alpha-1-acid glycoprotein	Q3SZR3	ORM1	183.68	31.19	6/6	3.80E+05	Propionamide (C)
A15	39/4.7	69.3/5.8	Albumin	P02769	ALB	235.24	29	16/16	1.11E+06	Propionamide (C)
		23.1/5.6	Alpha-1-acid glycoprotein	Q3SZR3	ORM1	198.56	38.61	9/9	4.48E+06	Propionamide (C), Deamidation (NQ)
A16	36/5.8									
A17	32.1/6	69.3/5.8	Albumin	P02769	ALB	112.78	7.58	4/4	4.57E+05	
A18	18.8/6.1									
AB1	97.1/4.2									
AB2	40.7/8.5									
AB3	21.9/5.9									
AB4	20.7/4.1									
AB5	21.5/8.7									

**TABLE 2:** Spots excised from HSF BSA resolved by 2DE and the proteoforms identified by LC-MS/MS with ≥3 identified peptides**.** All identified proteoforms were from the Bos taurus species. MW, molecular weight; pI, isoelectric point; PTM, post translational modification. Propionamide (C), oxidation (M), or deamidation (NQ) are not specified by amino acid residue as they are likely artifacts of sample preparation.

Spot ID	Observed MW (kDa)/pI	Theoretical MW (kDa)/pI	Protein identified	Accession number	Gene	Protein confidence score (−10LgP)	Sequence coverage (%)	Number of peptides/Unique peptides	Area of sample	PTM
B1	>250/5.8	69.3/5.8	Albumin	P02769	ALB	399.33	83.03	102/102	5.03E+07	Propionamide (C), Oxidation (M), Deamidation (NQ), Glycidamide adduct (L483)
B2	>250/5.8	69.3/5.8	Albumin	P02769	ALB	537.27	88.3	189/186	3.72E+08	Propionamide (C), Oxidation (M), Deamidation (NQ), Carboxyethyl (K548)
B3	>250/5.8	69.3/5.8	Albumin	P02769	ALB	521.15	82.87	166/166	2.24E+08	Propionamide (C), Oxidation (M), Deamidation (NQ), Glycidamide adduct (C223, L483), 2-amino-3-oxobutanoic acid (Y161)
B4	>250/5.8	69.3/5.8	Albumin	P02769	ALB	553.73	85.34	214/211	4.67E+08	Propionamide (C), Oxidation (M), Deamidation (NQ), Glycidamide adduct (C125, C223, L483), Dihydroxy (K489), Formylation (H402, K437), Carboxyethyl (K548), Carboxymethyl (K437)
		43.9/4.9	Keratin, type I cytoskeletal 19	P08728	KRT19	175.86	11.28	5/1	3.30E+04	
		57.7/7.1	Keratin, type II cytoskeletal 79	Q148H7	KRT79	144.37	5.61	3/1	1.79E+04	Deamidation (NQ)
B5	>250/5.8	69.3/5.8	Albumin	P02769	ALB	156.89	20.76	11/11	1.04E+06	Propionamide (C), Oxidation (M)
B6	151.1/5.8	69.3/5.8	Albumin	P02769	ALB	351.09	63.1	56/56	3.58E+07	Propionamide (C), Oxidation (M), Deamidation (NQ), Glycidamide Adduct (C223)
B7	69.3/5.6	69.3/5.8	Albumin	P02769	ALB	311.00	61.61	48/48	2.13E+07	Propionamide (C), Oxidation (M)
B8	69.3/5.6	69.3/5.8	Albumin	P02769	ALB	356.56	54.04	47/47	3.93E+07	Propionamide (C), Oxidation (M)
B9	69.3/5.7	69.3/5.8	Albumin	P02769	ALB	618.40	88.47	247/247	1.43E+09	Propionamide (C), Oxidation (M), Deamidation (NQ), Carboxyethyl (K548), Acetylation (D387), Methylation (K495), 2-amino-3-oxobutanoic acid (Y161), Carboxymethyl (K548)
B10	69.3/5.8	69.3/5.8	Albumin	P02769	ALB	596.15	90.44	237/236	8.42E+08	Propionamide (C), Oxidation (M), Deamidation (NQ), Glycidamide Adduct (C223, L483), Acetylation (S296), Formylation (K437, K495)
B11	69.3/5.8	69.3/5.8	Albumin	P02769	ALB	701.64	94.23	383/376	8.17E+09	Propionamide (C), Oxidation (M), Deamidation (NQ), Glycidamide Adduct (S310, L529), Methylation (E356, E406), Formylation (Q413), Acetylation (K75, S296, D387, K437), Carboxymethyl (K256, K548)
B12	69.3/5.8	69.3/5.8	Albumin	P02769	ALB	690.34	94.23	431/425	3.66E+09	Propionamide (C), Oxidation (M), Deamidation (NQ), Glycidamide Adduct (C125, L529), Dihydroxy (Y161), Acetylation (D387), Dehydration (T445), Carboxyethyl (K548), Phosphorylation (S512/T515)
B13	69.3/5.9	69.3/5.8	Albumin	P02769	ALB	691.75	91.76	396/395	3.88E+09	Propionamide (C), Oxidation (M), Deamidation (NQ), Glycidamide Adduct (C223, L529), Dihyhroxy (K489), Acetylation (K266, S296, D387), Methylation (E406, E488), Carboxyethyl (K587), 2-amino-3-oxobutanoic acid (Y161)
B14	69.3/5.9	69.3/5.8	Albumin	P02769	ALB	563.91	87.15	204/199	6.37E+08	Propionamide (C), Oxidation (M), Deamidation (NQ), Pyro-glu from Q (Q118)
B15	69.3/6	69.3/5.8	Albumin	P02769	1 SV	388.28	57.99	71/68	5.55E+07	Propionamide (C), Oxidation (M), Deamidation (NQ), 2-amino-3-oxobutanoic acid (Y161)
B16	69.3/6	69.3/5.8	Albumin	P02769	ALB	494.91	81.71	162/162	2.63E+08	Propionamide (C), Oxidation (M), Deamidation (NQ), Carboxyethyl (K100), Formylation (H402), Carboxymethyl (K437, K548)
		57.7/7.1	Keratin, type II cytoskeletal 79	Q148H7	KRT79	164.11	5.23	4/1	7.63E+04	Deamidation (NQ)
B17	69.3/6	69.3/5.8	Albumin	P02769	ALB	575.24	87.31	205/205	5.16E+08	Propionamide (C), Oxidation (M), Deamidation (NQ), Acetylation (S296), 2-amino-3-oxobutanoic acid (Y161)
B18	69.3/6.1	69.3/5.8	Albumin	P02769	ALB	498.87	83.53	158/155	1.72E+08	Propionamide (C), Oxidation (M), Deamidation (NQ), 2-amino-3-oxobutanoic acid (Y161)
		54.8/5.1	Keratin, type I cytoskeletal 10	P06394	KRT10	260.01	13.12	12/4	1.52E+05	Deamidation (NQ)
		57.7/7.1	Keratin, type II cytoskeletal 79	Q148H7	KRT79	182.52	5.61	4/1	4.48E+03	Deamidation (NQ)
B19	69.3/6.3	69.3/5.8	Albumin	P02769	ALB	332.98	64.25	51/51	2.02E+07	Propionamide (C), Oxidation (M), Deamidation (NQ)
		54.8/5.1	Keratin, type I cytoskeletal 10	P06394	KRT10	185.70	10.27	8/1	2.21E+04	
		57.7/7.1	Keratin, type II cytoskeletal 79	Q148H7	KRT79	135.43	3.36	3/2	2.54E+04	Deamidation (NQ)
B20	53.4/5.8	69.3/5.8	Albumin	P02769	ALB	426.30	81.55	107/104	8.18E+07	Propionamide (C), Oxidation (M), Deamidation (NQ), Glycidamide Adduct (D387, L483), Carboxyethyl (K100)
B21	53.4/5.9	69.3/5.8	Albumin	P02769	ALB	342.20	67.71	58/58	4.22E+07	Propionamide (C), Oxidation (M), Glycidamide Adduct (L483)
B22	53.4/5.9	69.3/5.8	Albumin	P02769	ALB	302.50	47.12	36/35	7.48E+06	Propionamide (C), Oxidation (M)
B23	45.1/5.6	69.3/5.8	Albumin	P02769	ALB	272.19	26.52	21/21	2.04E+06	Propionamide (C), Oxidation (M)
		57.7/7.1	Keratin, type II cytoskeletal 79	Q148H7	KRT79	140.04	5.61	3/1	2.57E+04	Deamidation (NQ)
B24	40.7/5.6	69.3/5.8	Albumin	P02769	ALB	307.79	57	35/35	1.59E+07	Propionamide (C), Oxidation (M)
		54.8/5.1	Keratin, type I cytoskeletal 10	P06394	KRT10	189.02	13.88	7/1	8.66E+03	Oxidation (M), Deamidation (NQ)
		57.7/7.1	Keratin, type II cytoskeletal 79	Q148H7	KRT79	135.66	5.61	3/1	2.22E+04	Deamidation (NQ)
B25	40.7/5.7	69.3/5.8	Albumin	P02769	ALB	91.91	7.58	4/4	2.97E+05	
B26	33.7/5.8	69.3/5.8	Albumin	P02769	ALB	354.39	67.05	51/50	1.36E+07	Propionamide (C), Oxidation (M)
B27	29/5.8	69.3/5.8	Albumin	P02769	ALB	248.45	23.56	16/16	6.95E+06	Propionamide (C), Oxidation (M)
B28	29/5.9	69.3/5.8	Albumin	P02769	ALB	250.33	32.95	20/20	4.36E+06	Propionamide (C), Oxidation (M)
B29	24.5/5.8	69.3/5.8	Albumin	P02769	ALB	282.34	25.7	14/14	2.58E+06	Propionamide (C)
		57.7/7.1	Keratin, type II cytoskeletal 79	Q148H7	KRT79	200.17	9.35	5/1	1.68E+05	Deamidation (NQ)
B30	24.5/5.9	69.3/5.8	Albumin	P02769	ALB	145.35	14.99	8/8	1.50E+06	Propionamide (C)
B31	24.5/6	69.3/5.8	Albumin	P02769	ALB	132.68	11.7	6/6	5.74E+05	
BB1	41.9/4.2									
BB2	43/8									
BB3	19.5/4.6									
BB4	19.4/8.2									
BB5	16.4/5.9	69.3/5.8	Albumin	P02769	ALB	160.96	12.52	7/7	7.38E+05	Propionamide (C)

## 4 Discussion

The ability to identify highly selective biomarkers and therapeutic targets, and to verify the purity of biologics, is significantly linked to the analytical methods available to achieving deep, comprehensive analysis of proteomes and their inherent proteoforms. However, the current state of proteomics is similar to the story of Pandora’s box, which is a metaphor for things that bring great trouble, but may also hold hope. Symbolically, the box represents curiosity and desire for knowledge that can lead to both consequences and outcomes. The evils inside the box can be seen as the challenges and difficulties of deep proteomic analyses, while the hope represents our optimism to overcome the challenges. The current “evils” in proteomics constitute an inability to definitively determine how many proteoforms are actually in a proteome because none of our analytical technologies have the ability to effectively detect and identify, let alone know, every proteoform. The “hope” lies in the power of current comprehensive analytical approaches such as iTDP, and the continued refinement, optimization, and development of analytical tools (and the willingness to recognize this necessity) to achieve ever more comprehensive analyses of proteomes at the critical level of proteoforms (Naryzhny, 2016; Zhan et al., 2019; Carbonara et al., 2021; 2023; Coorssen, 2023b; Coorssen and Padula, 2024).

Here, two different preparations of BSA were analysed, noting that both preparation methods are also used to isolate HSA for analytical and clinical applications. CEF, initially developed by Cohn and colleagues, is based on the solubility differences between albumin and other canonical plasma proteins in ethanol (Cohn et al., 1946; Cohn et al., 1950). Briefly, the temperature is reduced to −5°C while the concentration of ethanol increases from 8% to 40% and the pH (7.2–4.6) and ionic strength are adjusted (Raoufinia et al., 2016; Ma et al., 2020). Albumin precipitates in the higher ethanol concentration and lower pH, referred to as Fraction V. HSF involves heating plasma, to generally >60°C for 90 min to isolate albumin (Gonzalez et al., 2017; Ma et al., 2020). Notably, the two different preparation methods yield different proteoform profiles, with only two overlapping canonical protein species–albumin and bovine keratin, type I cytoskeletal 10. The list of identified ORF products is dominated by albumin, which has been processed into multiple proteoforms prior to fractionation of the starting blood material, during fractionation due to the conditions applied, and/or during sample preparation in which alkylation of cysteine, oxidation of methionine, and deamidation of asparagine/glutamine can occur (Figure 1; Tables 1, 2). Multiple proteoforms resulting from multimerization (higher MW, e.g., spots B1-B6; Table 2; Figure 1B) and cleavage (lower MW, e.g., spots A17 and B23-B31; Tables 1, 2; Figure 1) were identified, with some proteoforms apparently being the result of both (e.g., cleaved proteoforms associating). Being a globular protein, albumin folds in aqueous solutions to minimize conformational free energy and thus differences in purification methods, including exposure to organic solvents or thermal-induced fractionation (i.e., heat shock), can alter the conformation of BSA (Liu et al., 2010; Yoshikawa et al., 2012; Ma et al., 2020). A “high” degree of “purity” (>94%) is obtained when plasma is heated between 70°C and 75°C, however this is beyond the critical temperature of albumin at which structural changes are irreversible (Hoch and Chanutin, 1954; Park et al., 2018). Once albumin reaches its critical temperature, the loss of alpha helical character is not subsequently completely recovered resulting in the oligomerization of BSA molecules (Moriyama et al., 2008; Ma et al., 2020). Thermally induced multimerization likely explains the greater abundance of higher MW albumin species in spots B1-B6 of the HSF sample relative to the CEF. Other albumin proteoforms are the result of PTM that alter the charge of specific amino acids, causing a shift in the location of the proteoform within the horizontal pI dimension of the gel.

In addition to this albumin proteoform complexity, we observe multiple spots containing other co-purified proteoforms, either as single resolved proteoforms or co-localized with an albumin proteoform. Notably, vitamin D binding protein (VDPB) identified in spot A4 of the CEF sample. VDPB belongs to the albuminoid family–plasma proteins involved in fatty acid and hormone transport including albumin, α-fetoprotein and afamin (Bouillon et al., 2020). VDBP has three domains similar to albumin and shares similar physicochemical properties. While no co-purifying proteins in Fraction V have been reported in the literature, the data here indicate that VDBP co-precipitates with albumin. In contrast, alpha-1-acid glycoprotein identified in spots A14 and A15, has no structural similarities to albumin (Bteich, 2019). Had we not used 2DE, these identifications would represent somewhat of a conundrum, but as the pI and MW of the corresponding ORF products do not correlate with the location of the gel spots, these are clearly proteoforms or, more likely, fragments thereof (see, for example, (Sen et al., 2019). However, the low detected peptide counts for the ORF products were not sufficient to determine the nature of these proteoforms. Although using iTDP - the technology platform with the absolute highest resolving power for proteoforms – at the low total protein loads used here, there is still insufficient data for definitive answers. However, this also further highlights the common and dangerously speculative problem inherent to BUP, the assumption that the identification of even a single (unique) peptide automatically represents the presence of an intact canonical species. This then further emphasizes the need for the more fully comprehensive, routine analyses provided by iTDP, as well as the need for ongoing refinement and optimization of all analytical protocols (Carbonara et al., 2021; Coorssen and Padula, 2024). Similarly, with the typical use of under-loaded 1D SDS-PAGE gels, in which many more than one (un)related species is likely present in any single “band”, coupled with insensitive (outdated) staining methods for detection, it is perhaps not surprising to see manufacturers claim purities of >98–99% for purportedly isolated “proteins”. Using a more rigorous gel-based purity assessment, we estimated the purity of BSA isolated by CEF and HSF to be 57.8% and 49.7%, respectively (Supplementary Figure S5; Supplementary Table S3). Nonetheless, noting the low resolution of 1D gels and that small PTM (e.g., phosphorylation) would not significantly affect migration, there is the possibility that even what is defined as the “monomer” band in the gel (i.e., the expected canonical amino acid sequence) contains other proteoforms; thus, the purity estimates are essentially a best-case scenario and might actually be still somewhat lower in terms of the canonical species. This is important not only in terms of establishing sample “purity” (and what that really means) but in the fact that suppliers provide the product as the purified canonical species.

Clearly these commercial claims relative to the actual amount of the canonical species are insufficient regardless of the isolation strategy employed and call into serious question what it means to “purify” a protein (let alone one or more specific proteoforms; see Noaman et al., 2017 for detailed purity analysis of five different commercial protein isolates). Do we take this simply to mean that in a given preparation there are shared amino acid sequences or portions thereof, regardless of their abundance distribution and/or PTM? In particular, for biologics, it would seem that only a rigorous iTDP approach is sufficient to both effectively identify actual therapeutically active proteoform constituents and to ensure the true purity of the preparations supplied for clinical use. Failure to rigorously do so likely explains some of the recognized and dangerous side-effects of intravenous therapy with HSA biologics, including anaphylaxis (Pulimood and Park, 2000; Campos Munoz et al., 2024; Mayo Clinic, 2024). Notably, a key contraindication to the use of these HSA biologics is “hypersensitivity to any component in [the] albumin preparations … ‘; however, such preparations clearly do not comprise a single molecular entity, and thus the offending “components” are actually unknown. It will be important in the future to define and separate the clinically important proteoforms to yield more selective, safer therapeutics (Marie et al., 2013).

Overall, the results thus raise several important points: (1) if the “proteome” of a single “purified” protein is so complex (and dependent on sampling methods and sample handling), how can anything but unified protocols and iTDP analyses be justified for the analysis of whole proteome extracts from any native sample (2) what does it mean to “purify” a protein (i.e., often claiming near 100% purity)?; if the sample actually consists of a wide variety of proteoforms (let alone co-purifying species)?; (3) what is/are actual effective biomarkers if analyses assess only the generic canonical protein/ORF product (and depending on the analytical method, may even miss some if not all proteoforms); (4) what is/are the actual effective therapeutic species (and potentially dangerous species) in such generic isolates claimed to be of canonical species?; (5) what potentially important proteoforms are lost in analyses that utilize affinity “purification” of samples (e.g., plasma) and why is analysis of both the solute and retentate fractions not the insisted upon routine?; and (6) What does it mean to use such preparations as analytical standards and how do differences between preparations affect subsequent results (e.g. when used to calibrate the total protein in a sample for proteome analysis)? Failure to widely recognize and accept proteome complexity and the inherent need to carry out analyses at the level of proteoforms rather than canonical ORF products has likely delayed the identification and validation of effective biomarkers and new, more selective drugs and therapeutic targets by two decades or more. In this post-proteogenomic era, there is no further excuse for not engaging in the deep, truly comprehensive analysis of proteomes that will provide the much-needed positive changes in biomarker and drug development (Carbonara et al., 2021; Coorssen and Padula, 2024).

In considering this complexity, we do not believe that the results presented here likely convey the actual *in vivo*/*in situ* complexity of the albuminome, or any (sub)proteome or interactome, but rather that they emphasize the need for more critical consideration of the specifics of sample collection, processing, handling, storage, and analysis. Furthermore, the data again emphasize that BUP analyses simply cannot provide the critical details necessary to genuinely understand proteome complexity at the level of proteoforms and their quantification (even in supposedly “pure” protein isolates).

The limitations of the current study are thus common to essentially all proteomic studies to date, although these are particularly complicated by working with a blood product (Coorssen and Padula, 2024): 1) a commercial preparation, likely derived from the combined blood of dozens or more individuals; 2) preparation method – although chosen to avoid the shortcomings associated with the Cohn method, heat shock has its own shortcomings in terms of the objective; 3) the likely loss of interacting proteoforms (beyond those covalently or otherwise tightly bound) cannot be discounted with either process; 4) loss of native structure during commercial processing and/or reduction and alkylation here can have influenced the results; 5) cannot differentiate between genuine interactors and co-purifying proteoforms (i.e., “contaminants”); and 6) in those instances in which a proteoform was identified based on pI and/or MW but a specific, corresponding modified proteotypic peptide was not isolated, while we have confidence in the isolation of a proteoform we cannot be completely certain of its specific chemical characteristics (e.g., PTM, isoform, mutation, adducts) although the ORF product identifications are accurate based on current databases. Nonetheless, having used iTDP, we have more information than available by any other method and can be certain of size and charge variations – as well as proteoform monomers and oligomers – all physicochemical characteristics that influence molecular interactions.

One must consider what would it mean to effectively assess the albumin (or any) interactome, and how inherent issues likely impact, to varying extents, any such analyses of proteoform molecular interactions? The issues begin with sampling. First, any blood drawn with smaller gauge needles results in some lysis of platelets and circulating cells thus (i) contaminating the blood sample with myriad proteoforms that the constituent albumin is unlikely to normally ever be exposed to but could bind; and (ii) releasing a host of proteases that, again, are unlikely normally to be so freely present in native circulating blood. Thus, second, were broad spectrum protease inhibitors added, and preferably kinase and phosphatase inhibitors as well (Butt and Coorssen, 2005; Wright et al., 2014a). Third, how long was the blood left and at what temperature before further processing? Regarding the commercial isolates used in this study, prior to HSF to isolate BSA, the serum was subjected to pH < 5 and a temperature in excess of 65°C for a least 3 h, for the purpose of inactivating viral pathogens. Fourth, if the sample is stored either before or after further processing, was it appropriately snap frozen or simply placed in a freezer to slowly crystallize? Fifth, were samples aliquoted so that there was never more than a single freeze-thaw cycle (Jeffs et al., 2019)? Sixth, could any of the other processing/handling/storage steps have resulted in losses of proteoforms of the “protein” of interest or otherwise affected their structure or physicochemical properties and thus the native molecular interactions (i.e., causing loss of bound species or failure to quantitatively account for proteoforms)? Seventh, could the analytical process used have caused displacement/unbinding of interacting species that would result in either their loss from the analysis or their identification as a co-purifying/contaminating species rather than an interactor? Eighth, has the analysis used taken into account all proteoforms of the “protein” of interest as such information is the key to understanding complexity and the specificity it imbues to interactomes. Ninth, can the analytical methods used (i) distinguish between interactors and co-purifying species and (ii) differentiate weak/transient vs. strong (i.e., covalent) interactors?

Although previous studies have used different approaches to define an HSA albuminome/interactome, (e.g., 1D gel electrophoresis, crosslinking, LC-MS/MS), the quality of canonical protein identifications varied as did overlap between the datasets. Nonetheless, all three co-purifying species identified here have in one or more other studies been identified as “interacting” with HSA, either as canonical proteins or variants thereof (Zhou et al., 2004; Gundry et al., 2007; Scumaci et al., 2011; Liu et al., 2017; Hauser et al., 2018). With regard to the data here, the question thus arises as to what constitutes an interacting vs. a co-purifying species, or an artifact of the isolation and/or analytical methods used? To genuinely understand the native albumin interactome or albuminome, a clear distinction between these terms should be made. Considering the complexity observed even in purified samples, perhaps the term “albuminome” would best apply to the actual collection of albumin proteoforms in any given sample. While we recognize that *circulating* blood is the real interest in this regard, it is also clear that methods of sampling, purification, and sample handling have effects that can no longer be ignored. By definition, then, the albumin interactome would constitute any molecular species capable of interacting with constituents of the albuminome, even if only transiently; here the interest is in proteoforms that interact with any proteoform of albumin, rather than drugs or other molecular species found in *circulating* blood. These more specific definitions thus also enable more definitive identification of bound/interacting vs. co-purifying/contaminating species. However, there nonetheless remains the question of how well *in vitro* interaction/affinity studies represent the complex reality that is *circulating* blood *in vivo*. That is, while powerful in their own right, it is difficult if not impossible for reductionist *in vitro* approaches to fully capture the complexity of native systems; the possibility of missing critical interactions or identifying spurious interactions must always be considered and effectively controlled for (as best possible).

To summarize, considering the issue from a systems perspective, here we carried out a proof-of-principle study - an initial assessment, addressing albumin isolates as proteomes rather than generic bulk entities. The aim is to initiate a more holistic consideration of what constitutes the “albuminome” as a model for the more systematic analysis of (sub)proteomes and the molecular interactions (i.e., interactomes) inherent to them. If systems as “simple” as a supposedly purified protein are in reality already as complex as revealed by these initial analyses, how can anything other than iTDP be considered sufficient to analyse native proteomes (Naryzhny, 2016; Naryzhny, 2024; Zhan et al., 2019; Coorssen and Padula, 2024). Simply, the identification of effective, selective biomarkers and therapeutics cannot continue in the same old manner that has been practiced for decades (D’Silva et al., 2020; Sen et al., 2021). To achieve this will require the continued refinement and optimized coupling of 2D gel electrophoresis, liquid chromatography, and tandem mass spectrometry, improved sensitivity overall, and open search algorithms to more definitively identify spectra of PTM-containing peptides, and assign the nature and site of the modification (Carbonara et al., 2021; Polasky et al., 2023; Coorssen and Padula, 2024). It is thus also noteworthy that gel-based electrophoretic methods have a long history of use for identifying potential biomarkers (Issaq and Veenstra, 2007), that a curated database of human disease associated PTMs is readily accessible (Xu et al., 2018), and that efforts are already underway to at least begin addressing therapeutic selectivity at the level of isoforms (Kjer-Hansen et al., 2024).

The iTDP analytical approach would thus appear to be the most logical way forward to characterise, as best possible, the entirety of a proteome and therefore serve as an effective tool in experimental design, refinement of computational/mathematical models of disease states, and for the discovery/design, refinement, and validation of truly selective therapeutics and biomarkers.

## Data Availability

The datasets presented in this study can be found in online repositories (Perez-Riverol et al., 2022). The names of the repository/repositories and accession number(s) can be found below: https://www.ebi.ac.uk/pride/archive/, PXD056316.
