# Supplementary material for: Protein “purity,” proteoforms, and the albuminome: critical observations on proteome and systems complexity
Source: Front Cell Dev Biol. 2024 Dec 10;12:1504098. doi: 10.3389/fcell.2024.1504098 (PMC11666697; doi:10.3389/fcell.2024.1504098)
Supplement: Supplementary file 1 [file DataSheet1.docx]

Supplementary Material

**A**

| 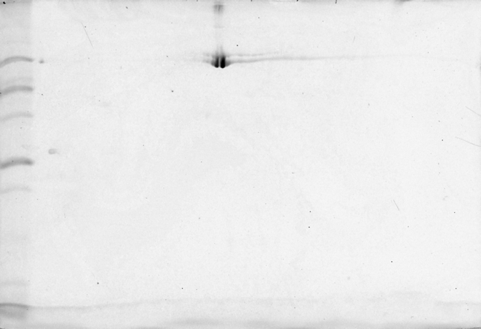  **D** | 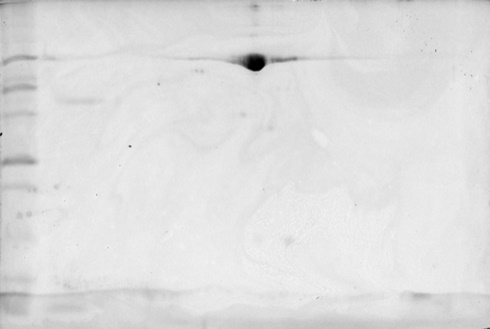  **B** | 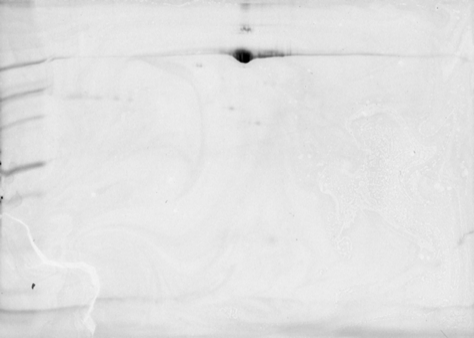  **C** |
| --- | --- | --- |
| 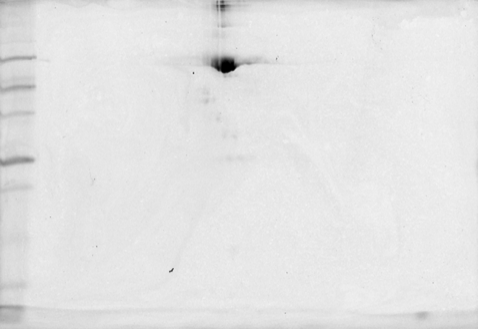 | 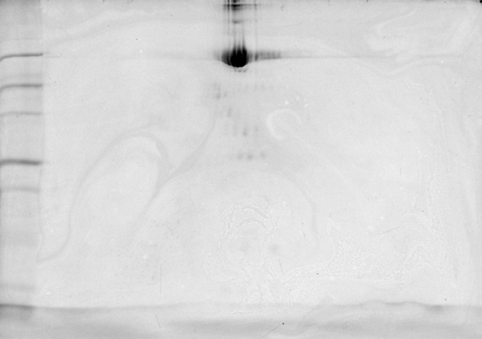  **E** | 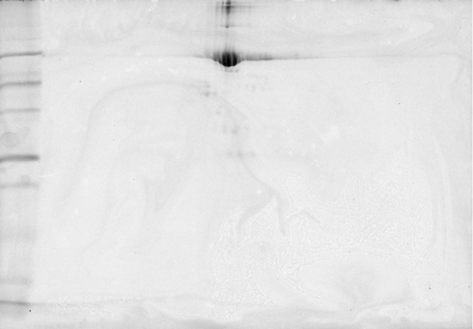  **F** |

Supplementary Figure S1. Gel images of resolved bovine serum albumin stained for total proteoform detection with cCBB. Raw 2DE gel images of resolved BSA samples purified by cold-ethanol fractionation (A-C) or heat-shock fractionation (D-F). Gels were stained for total proteoform detection with cCBB only (A & D), stained for glycoproteoforms with a Glycoprotein Staining Kit then total proteoform detection with cCBB (B & E), and stained for phosphoproteoforms with Pro-Q Diamond then then total proteoform detection with cCBB (C & F). Gel A was excluded from analyses and subsequent experiments as it was significantly underloaded relative to the other gels, as indicated by total fluorescence signal.

| 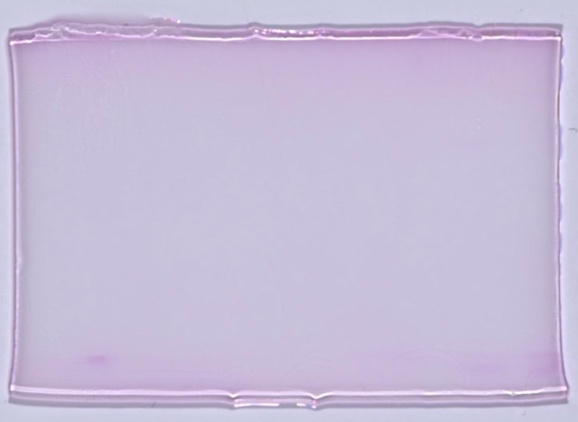  **A** | 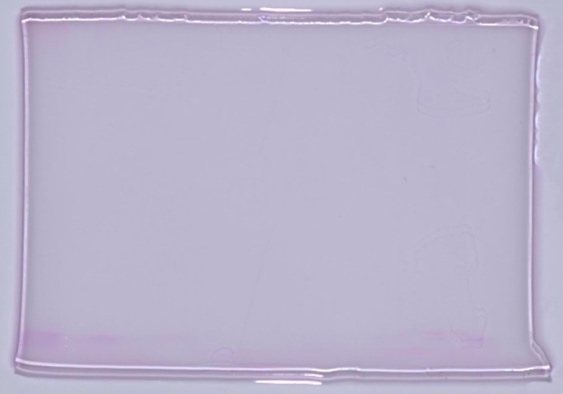  **B** |
| --- | --- |

Supplementary Figure S2. Gel images of resolved BSA stained for glycoproteoforms. Raw 2DE gel images of resolved BSA samples purified by cold-ethanol fractionation (A) or heat-shock fractionation (B) stained with Pierce™ Glycoprotein Staining Kit, according to manufacturer’s protocols.

**B**

| 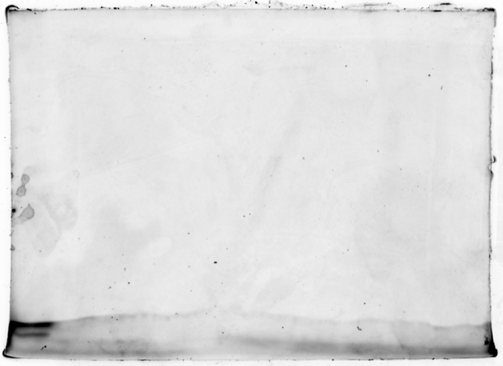  **A** | 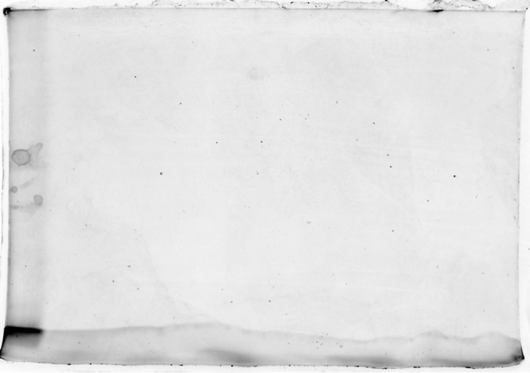 |
| --- | --- |

Supplementary Figure S3. Gel images of resolved BSA stained for phosphoproteoforms. Raw 2DE gel images of resolved BSA samples purified by cold-ethanol fractionation (A) or heat-shock fractionation (B) stained with Invitrogen^TM^ Pro-Q^TM^ Diamond, according to manufacturer’s protocols.


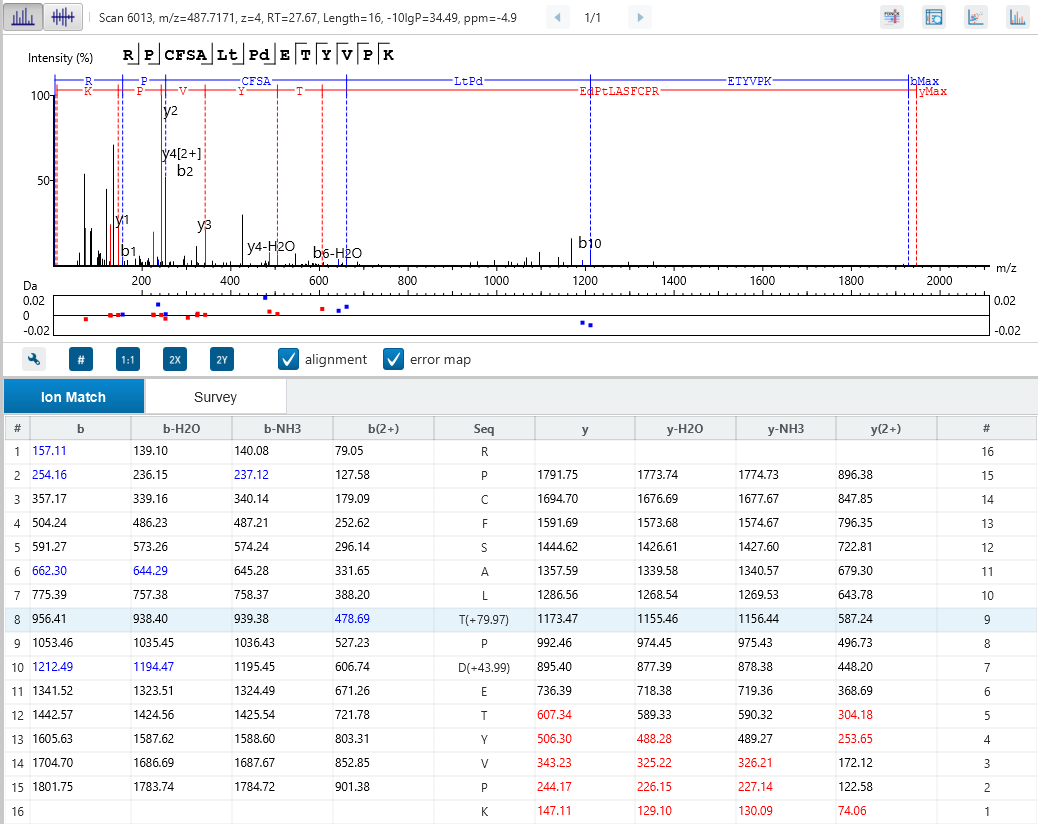


**Supplementary Figure S4. Phosphorylated peptide detected in spot B12.** The spectrum supports the presence of a phosphorylated peptide, however, the lack of a b-ion or y-ion of the modified residue to specifically localise the modification makes the assignment ambiguous. The doubly charged b-ion (478.69) could be due to the fact that there is an arginine at the N-terminal of the peptide that is doubly charged. However, this is a missed cleavage and is an example of trypsin not cutting if the next amino acid is Proline (J Proteome Res. 2008. 7(1): 300-5.). The presence of Proline in a sequence can have negative effects on fragmentation and the sequence ions are normally greatly reduced in intensity following a Proline (Int J Mass Spectrom. 2011. 308(1): 89-97.). The presence of three prolines is likely disrupting fragment intensities and thus why the y sequence ions are not detectable after y5.

**Table S1. Spots excised from cold ethanol fractionated BSA resolved by 2DE and the proteoforms identified by LC-MS/MS.** All identified proteoforms are from the *Bos taurus* species. MW, molecular weight; pI, isoelectric point; PTM, post translational modification.

| **Spot ID** | **Observed MW (kDa)/pI** | **Theoretical MW (kDa)/pI** | **Protein Identified** | **Accession Number** | **Gene** | **Protein Confidence Score  (-10lgP)** | **Sequence Coverage (%)** | **Number of Peptides / Unique Peptides** | **Area of Sample** | **PTM** |
| --- | --- | --- | --- | --- | --- | --- | --- | --- | --- | --- |
| A1 | >250 / 5.9 | 69.3 / 5.8 | Albumin | P02769 | ALB | 357.27 | 72.65 | 68 / 68 | 2.02E+07 | Propionamide (C), Oxidation (M), Deamidation (NQ) |
| A2 | 105.8 / 5.9 | 69.3 / 5.8 | Albumin | P02769 | ALB | 266.56 | 27.84 | 19 / 19 | 7.71E+06 | Propionamide (C), Oxidation (M), |
| A3 | 103.9 / 6 | 69.3 / 5.8 | Albumin | P02769 | ALB | 401.42 | 78.42 | 89 / 89 | 7.71E+07 | Propionamide (C), Oxidation (M), Deamidation (NQ), Glycidamide Adduct (L483), Carboxyethyl (K100, K437) |
|  |  | 81.8 / 5.8 | Junction plakoglobin | Q8SPJ1 | JUP | 58.29 | 1.88 | 1 / 1 | 5.76E+03 | Oxidation (M) |
| A4 | 53.8 / 5.5 | 69.3 / 5.8 | Albumin | P02769 | ALB | 254.91 | 36.08 | 18 / 18 | 2.48E+06 | Propionamide (C), Oxidation (M), Deamidation (NQ) |
|  |  | 53.3 / 5.4 | Vitamin D-binding protein | Q3MHN5 | GC | 195.26 | 18.78 | 8 / 8 | 1.68E+06 | Propionamide (C) |
|  |  | 81.8 / 5.8 | Junction plakoglobin | Q8SPJ1 | JUP | 76.12 | 1.88 | 1 / 1 | 1.13E+04 | Oxidation (M) |
| A5 | 64.2 / 5.8 | 69.3 / 5.8 | Albumin | P02769 | ALB | 556.65 | 85.17 | 206 / 206 | 4.65E+08 | Propionamide (C), Oxidation (M), Deamidation (NQ), Glycidamide Adduct (C125, C223, L483), Formylation (H402), Carboxyethyl (C437, K548) |
| A6 | 64.2 / 5.9 | 69.3 / 5.8 | Albumin | P02769 | ALB | 713.51 | 92.09 | 352 / 350 | 5.95E+09 | Propionamide (C), Oxidation (M), Deamidation (NQ), Glycidamide Adduct (C125, L529), Dehydration (T550), Acetylation (S296, D387), Formylation (R508) |
| A7 | 64.2 / 5.9 | 69.3 / 5.8 | Albumin | P02769 | ALB | 730.13 | 92.09 | 427 / 421 | 9.62E+09 | Propionamide (C), Oxidation (M), Deamidation (NQ), Methylation (K318), Carboxylation (K548), Acetylation (D387), Glycidamide Adduct (C223, L529), Formylation (Q413) Carboxyethyl (K100) |
| A8 | 64.2 / 6 | 69.3 / 5.8 | Albumin | P02769 | ALB | 702.09 | 92.75 | 369 / 365 | 4.71E+09 | Propionamide (C), Oxidation (M), Deamidation (NQ), Glycidamide Adduct (C125, C223), Acetylation (S296, D38), Dehydration (T550), Carboxyethyl (K100), 2-amino-3-oxobutanoic acid (Y161), Dehydration (T550), Amidation (F43)) |
| A9 | 64.2 / 6 | 69.3 / 5.8 | Albumin | P02769 | ALB | 564.59 | 87.64 | 206 / 205 | 8.22E+08 | Propionamide (C), Oxidation (M), Deamidation (NQ), Glycamide Adduct (Q118, C125, C223, Dihydroxy (Y161), Acetylation (S296), Carboxyethyl (K548), 2-amino-3-oxobutanoic acid (Y161) |
| A10 | 64.2 / 6.1 | 69.3 / 5.8 | Albumin | P02769 | ALB | 546.24 | 82.54 | 166 / 164 | 3.41E+08 | Propionamide (C), Oxidation (M), Deamidation (NQ), Glycamide Adduct (C125), Acetylation (Q118), Formylation (H402), 2-amino-3-oxobutanoic acid (Y161) |
|  |  | 54.8 / 5.1 | Keratin, type I cytoskeletal 10 | P06394 | KRT10 | 154.87 | 6.46 | 3 / 1 | 1.33E+04 |  |
|  |  | 57.7 / 7.1 | Keratin, type II cytoskeletal 79 | Q148H7 | KRT79 | 31.15 | 2.62 | 1 / 1 | 4.59E+03 |  |
|  |  | 17.2 / 6.2 | Glycosylation-dependent cell adhesion molecule 1 | P80195 | GLYCAM1 | 98.06 | 15.69 | 1 / 1 | 2.86E+04 |  |
| A11 | 64.2 / 6.2 | 69.3 / 5.8 | Albumin | P02769 | ALB | 302.51 | 49.59 | 44 / 44 | 1.39E+07 | Propionamide (C), Oxidation (M), Deamidation (NQ) |
| A12 | 64.2 / 3.1 | 69.3 / 5.8 | Albumin | P02769 | ALB | 66.44 | 11.53 | 9 / 9 | 4.29E+05 | Propionamide (C) |
| A13 | 39.6 / 3.3 | 69.3 / 5.8 | Albumin | P02769 | ALB | 389.12 | 75.78 | 57 / 57 | 1.51E+07 | Propionamide (C), Oxidation (M) |
| A14 | 39.2 / 4.2 | 69.3 / 5.8 | Albumin | P02769 | ALB | 346.58 | 75.29 | 48 / 47 | 1.68E+07 | Propionamide (C), Oxidation (M) |
|  |  | 23.1 / 5.6 | Alpha-1-acid glycoprotein | Q3SZR3 | ORM1 | 183.68 | 31.19 | 6 / 6 | 3.80E+05 | Propionamide (C) |
| A15 | 39 / 4.7 | 69.3 / 5.8 | Albumin | P02769 | ALB | 235.24 | 29 | 16 / 16 | 1.11E+06 | Propionamide (C) |
|  |  | 23.1 / 5.6 | Alpha-1-acid glycoprotein | Q3SZR3 | ORM1 | 198.56 | 38.61 | 9 / 9 | 4.48E+06 | Propionamide (C), Deamidation (NQ) |
| A16 | 36 / 5.8 | 69.3 / 5.8 | Albumin | P02769 | ALB | 73.46 | 4.28 | 2 / 2 | 1.57E+05 |  |
| A17 | 32.1 / 6 | 69.3 / 5.8 | Albumin | P02769 | ALB | 112.78 | 7.58 | 4 / 4 | 4.57E+05 |  |
| A18 | 18.8 / 6.1 |  |  |  |  |  |  |  |  |  |
| AB1 | 97.1 / 4.2 |  |  |  |  |  |  |  |  |  |
| AB2 | 40.7 / 8.5 |  |  |  |  |  |  |  |  |  |
| AB3 | 21.9 / 5.9 |  |  |  |  |  |  |  |  |  |
| AB4 | 20.7 / 4.1 |  |  |  |  |  |  |  |  |  |
| AB5 | 21.5 / 8.7 |  |  |  |  |  |  |  |  |  |

**Table S2. Spots excised from heat shock fractionated BSA resolved by 2DE and the proteoforms identified by LC-MS/MS.** All identified proteoforms are from the *Bos taurus* species. MW, molecular weight; pI, isoelectric point; PTM, post translational modification.

| **Spot ID** | **Observed MW (kDa)/pI** | **Theoretical MW (kDa)/pI** | **Protein Identified** | **Accession Number** | **Gene** | **Protein Confidence Score  (-10lgP)** | **Sequence Coverage (%)** | **Number of Peptides / Unique Peptides** | **Area of Sample** | **PTM** |
| --- | --- | --- | --- | --- | --- | --- | --- | --- | --- | --- |
| B1 | >250 / 5.8 | 69.3 / 5.8 | Albumin | P02769 | ALB | 399.33 | 83.03 | 102 / 102 | 5.03E+07 | Propionamide (C), Oxidation (M), Deamidation (NQ), Glycidamide adduct (L483) |
| B2 | >250 / 5.8 | 69.3 / 5.8 | Albumin | P02769 | ALB | 537.27 | 88.3 | 189 / 186 | 3.72E+08 | Propionamide (C), Oxidation (M), Deamidation (NQ), Carboxyethyl (K548) |
|  |  | 41.8 / 5.3 | Actin, cytoplasmic 1/2 | P60712 / P63258 | ACTB / ACTG1 | 140.29 | 9.07 | 2 / 2 | 4.03E+05 |  |
|  |  | 81.8 / 5.8 | Junction plakoglobin | Q8SPJ1 | JUP | 136.67 | 3.62 | 2 / 2 | 5.96E+04 |  |
|  |  | 71.2 / 5.4 | Heat shock cognate 71 kDa protein | P19120 | HSPA8 | 138.61 | 5.23 | 2 / 2 | 1.90E+05 |  |
| B3 | >250 / 5.8 | 69.3 / 5.8 | Albumin | P02769 | ALB | 521.15 | 82.87 | 166 / 166 | 2.24E+08 | Propionamide (C), Oxidation (M), Deamidation (NQ), Glycidamide adduct (C223, L483), 2-amino-3-oxobutanoic acid (Y161) |
| B4 | >250 / 5.8 | 69.3 / 5.8 | Albumin | P02769 | ALB | 553.73 | 85.34 | 214 / 211 | 4.67E+08 | Propionamide (C), Oxidation (M), Deamidation (NQ), Glycidamide adduct (C125, C223, L483), Dihydroxy (K489), Formylation (H402, K437), Carboxyethyl (K548), Carboxymethyl (K437) |
|  |  | 43.9 / 4.9 | Keratin, type I cytoskeletal 19 | P08728 | KRT19 | 175.86 | 11.28 | 5 / 1 | 3.30E+04 |  |
|  |  | 57.7 / 7.1 | Keratin, type II cytoskeletal 79 | Q148H7 | KRT79 | 144.37 | 5.61 | 3 / 1 | 1.79E+04 | Deamidation (NQ) |
|  |  | 81.8 / 5.8 | Junction plakoglobin | Q8SPJ1 | JUP | 132.07 | 3.09 | 2 / 2 | 2.39E+04 | Oxidation (M) |
| B5 | >250 / 5.8 | 69.3 / 5.8 | Albumin | P02769 | ALB | 156.89 | 20.76 | 11 / 11 | 1.04E+06 | Propionamide (C), Oxidation (M) |
| B6 | 151.1 / 5.8 | 69.3 / 5.8 | Albumin | P02769 | ALB | 351.09 | 63.1 | 56 / 56 | 3.58E+07 | Propionamide (C), Oxidation (M), Deamidation (NQ), Glycidamide Adduct (C223) |
| B7 | 69.3 / 5.6 | 69.3 / 5.8 | Albumin | P02769 | ALB | 311.00 | 61.61 | 48 / 48 | 2.13E+07 | Propionamide (C), Oxidation (M) |
| B8 | 69.3 / 5.6 | 69.3 / 5.8 | Albumin | P02769 | ALB | 356.56 | 54.04 | 47 / 47 | 3.93E+07 | Propionamide (C), Oxidation (M) |
| B9 | 69.3 / 5.7 | 69.3 / 5.8 | Albumin | P02769 | ALB | 618.40 | 88.47 | 247 / 247 | 1.43E+09 | Propionamide (C), Oxidation (M), Deamidation (NQ), Carboxyethyl (K548), Acetylation (D387), Methylation (K495), 2-amino-3-oxobutanoic acid (Y161), Carboxymethyl (K548) |
| B10 | 69.3 / 5.8 | 69.3 / 5.8 | Albumin | P02769 | ALB | 596.15 | 90.44 | 237 / 236 | 8.42E+08 | Propionamide (C), Oxidation (M), Deamidation (NQ), Glycidamide Adduct (C223, L483), Acetylation (S296), Formylation (K437, K495) |
| B11 | 69.3 / 5.8 | 69.3 / 5.8 | Albumin | P02769 | ALB | 701.64 | 94.23 | 383 / 376 | 8.17E+09 | Propionamide (C), Oxidation (M), Deamidation (NQ), Glycidamide Adduct (S310, L529), Methylation (E356, E406), Formylation (Q413), Acetylation (K75, S296, D387, K437), Carboxymethyl (K256, K548) |
| B12 | 69.3 / 5.8 | 69.3 / 5.8 | Albumin | P02769 | ALB | 690.34 | 94.23 | 431 / 425 | 3.66E+09 | Propionamide (C), Oxidation (M), Deamidation (NQ), Glycidamide Adduct (C125, L529), Dihydroxy (Y161), Acetylation (D387), Dehydration (T445), Carboxyethyl (K548), Phosphorylation (S512/T515) |
| B13 | 69.3 / 5.9 | 69.3 / 5.8 | Albumin | P02769 | ALB | 691.75 | 91.76 | 396 / 395 | 3.88E+09 | Propionamide (C), Oxidation (M), Deamidation (NQ), Glycidamide Adduct (C223, L529), Dihyhroxy (K489), Acetylation (K266, S296, D387), Methylation (E406, E488), Carboxyethyl (K587), 2-amino-3-oxobutanoic acid (Y161) |
| B14 | 69.3 / 5.9 | 69.3 / 5.8 | Albumin | P02769 | ALB | 563.91 | 87.15 | 204 / 199 | 6.37E+08 | Propionamide (C), Oxidation (M), Deamidation (NQ), Pyro-glu from Q (Q118), |
| B15 | 69.3 / 6 | 69.3 / 5.8 | Albumin | P02769 | 1 SV | 388.28 | 57.99 | 71 / 68 | 5.55E+07 | Propionamide (C), Oxidation (M), Deamidation (NQ), 2-amino-3-oxobutanoic acid (Y161) |
| B16 | 69.3 / 6 | 69.3 / 5.8 | Albumin | P02769 | ALB | 494.91 | 81.71 | 162 / 162 | 2.63E+08 | Propionamide (C), Oxidation (M), Deamidation (NQ), Carboxyethyl (K100), Formylation (H402), Carboxymethyl (K437, K548) |
|  |  | 57.7 / 7.1 | Keratin, type II cytoskeletal 79 | Q148H7 | KRT79 | 164.11 | 5.23 | 4 / 1 | 7.63E+04 | Deamidation (NQ) |
|  |  | 81.8 / 5.8 | Junction plakoglobin | Q8SPJ1 | JUP | 98.10 | 3.09 | 2 / 2 | 4.55E+04 | Oxidation (M) |
| B17 | 69.3 / 6 | 69.3 / 5.8 | Albumin | P02769 | ALB | 575.24 | 87.31 | 205 / 205 | 5.16E+08 | Propionamide (C), Oxidation (M), Deamidation (NQ), Acetylation (S296), 2-amino-3-oxobutanoic acid (Y161) |
|  |  | 17.2 / 6.2 | Glycosylation-dependent cell adhesion molecule 1 | P80195 | GLYCAM1 | 144.10 | 20.26 | 2 / 2 | 1.33E+06 | Deamidation (NQ) |
| B18 | 69.3 / 6.1 | 69.3 / 5.8 | Albumin | P02769 | ALB | 498.87 | 83.53 | 158 / 155 | 1.72E+08 | Propionamide (C), Oxidation (M), Deamidation (NQ), 2-amino-3-oxobutanoic acid (Y161) |
|  |  | 54.8 / 5.1 | Keratin, type I cytoskeletal 10 | P06394 | KRT10 | 260.01 | 13.12 | 12 / 4 | 1.52E+05 | Deamidation (NQ) |
|  |  | 57.7 / 7.1 | Keratin, type II cytoskeletal 79 | Q148H7 | KRT79 | 182.52 | 5.61 | 4 / 1 | 4.48E+03 | Deamidation (NQ) |
| B19 | 69.3 / 6.3 | 69.3 / 5.8 | Albumin | P02769 | ALB | 332.98 | 64.25 | 51 / 51 | 2.02E+07 | Propionamide (C), Oxidation (M), Deamidation (NQ) |
|  |  | 54.8 / 5.1 | Keratin, type I cytoskeletal 10 | P06394 | KRT10 | 185.70 | 10.27 | 8 / 1 | 2.21E+04 |  |
|  |  | 57.7 / 7.1 | Keratin, type II cytoskeletal 79 | Q148H7 | KRT79 | 135.43 | 3.36 | 3 / 2 | 2.54E+04 | Deamidation (NQ) |
| B20 | 53.4 / 5.8 | 69.3 / 5.8 | Albumin | P02769 | ALB | 426.30 | 81.55 | 107 / 104 | 8.18E+07 | Propionamide (C), Oxidation (M), Deamidation (NQ), Glycidamide Adduct (D387, L483), Carboxyethyl (K100) |
|  |  | 57.7 / 7.1 | Keratin, type II cytoskeletal 79 | Q148H7 | KRT79 | 113.96 | 3.93 | 2 / 1 | 1.14E+04 | Deamidation (NQ) |
| B21 | 53.4 / 5.9 | 69.3 / 5.8 | Albumin | P02769 | ALB | 342.20 | 67.71 | 58 / 58 | 4.22E+07 | Propionamide (C), Oxidation (M), Glycidamide Adduct (L483) |
| B22 | 53.4 / 5.9 | 69.3 / 5.8 | Albumin | P02769 | ALB | 302.50 | 47.12 | 36 / 35 | 7.48E+06 | Propionamide (C), Oxidation (M) |
| B23 | 45.1 / 5.6 | 69.3 / 5.8 | Albumin | P02769 | ALB | 272.19 | 26.52 | 21 / 21 | 2.04E+06 | Propionamide (C), Oxidation (M) |
|  |  | 57.7 / 7.1 | Keratin, type II cytoskeletal 79 | Q148H7 | KRT79 | 140.04 | 5.61 | 3 / 1 | 2.57E+04 | Deamidation (NQ) |
| B24 | 40.7 / 5.6 | 69.3 / 5.8 | Albumin | P02769 | ALB | 307.79 | 57 | 35 / 35 | 1.59E+07 | Propionamide (C), Oxidation (M) |
|  |  | 54.8 / 5.1 | Keratin, type I cytoskeletal 10 | P06394 | KRT10 | 189.02 | 13.88 | 7 / 1 | 8.66E+03 | Oxidation (M), Deamidation (NQ) |
|  |  | 57.7 / 7.1 | Keratin, type II cytoskeletal 79 | Q148H7 | KRT79 | 135.66 | 5.61 | 3 / 1 | 2.22E+04 | Deamidation (NQ) |
|  |  | 81.8 / 5.8 | Junction plakoglobin | Q8SPJ1 | JUP | 75.60 | 1.88 | 1 / 1 | 8.39E+03 | Oxidation (M) |
| B25 | 40.7 / 5.7 | 69.3 / 5.8 | Albumin | P02769 | ALB | 91.91 | 7.58 | 4 / 4 | 2.97E+05 |  |
| B26 | 33.7 / 5.8 | 69.3 / 5.8 | Albumin | P02769 | ALB | 354.39 | 67.05 | 51 / 50 | 1.36E+07 | Propionamide (C), Oxidation (M) |
| B27 | 29 / 5.8 | 69.3 / 5.8 | Albumin | P02769 | ALB | 248.45 | 23.56 | 16 / 16 | 6.95E+06 | Propionamide (C), Oxidation (M) |
|  |  | 57.7 / 7.1 | Keratin, type II cytoskeletal 79 | Q148H7 | KRT79 | 108.44 | 3.36 | 2 / 1 | 1.43E+04 | Deamidation (NQ) |
| B28 | 29 / 5.9 | 69.3 / 5.8 | Albumin | P02769 | ALB | 250.33 | 32.95 | 20 / 20 | 4.36E+06 | Propionamide (C), Oxidation (M) |
| B29 | 24.5 / 5.8 | 69.3 / 5.8 | Albumin | P02769 | ALB | 282.34 | 25.7 | 14 / 14 | 2.58E+06 | Propionamide (C) |
|  |  | 57.7 / 7.1 | Keratin, type II cytoskeletal 79 | Q148H7 | KRT79 | 200.17 | 9.35 | 5 / 1 | 1.68E+05 | Deamidation (NQ) |
|  |  | 81.8 / 5.8 | Junction plakoglobin | Q8SPJ1 | JUP | 124.65 | 3.09 | 2 / 2 | 8.39E+04 | Oxidation (M) |
| B30 | 24.5 / 5.9 | 69.3 / 5.8 | Albumin | P02769 | ALB | 145.35 | 14.99 | 8 / 8 | 1.50E+06 | Propionamide (C) |
| B31 | 24.5 / 6 | 69.3 / 5.8 | Albumin | P02769 | ALB | 132.68 | 11.7 | 6 / 6 | 5.74E+05 |  |
| BB1 | 41.9 / 4.2 |  |  |  |  |  |  |  |  |  |
| BB2 | 43 / 8 |  |  |  |  |  |  |  |  |  |
| BB3 | 19.5 / 4.6 |  |  |  |  |  |  |  |  |  |
| BB4 | 19.4 / 8.2 |  |  |  |  |  |  |  |  |  |
| BB5 | 16.4 / 5.9 | 69.3 / 5.8 | Albumin | P02769 | ALB | 160.96 | 12.52 | 7 / 7 | 7.38E+05 | Propionamide (C) |

|  |  |
| --- | --- |

**Figure S5.** Gel-based purity assessment of bovine serum albumin (BSA) isolated by cold ethanol fractionation (A) and heat shock fractionation (B). Total protein load is indicated above each lane.

**Table S3:** Purity assessments of bovine serum albumin (BSA) isolated by cold ethanol fractionation (CEF) and heat shock fractionation (HSF).

| **Protein** | **Product number** | **Lot number** | **Supplier-specified purity** | **Gel-based purity assessment (protein monomer band % of total lane signal)** |
| --- | --- | --- | --- | --- |
| CEF BSA | A7517 | SLCM2607 | Not specified | 57.78% |
| HSF BSA | A8022 | SLBC3033V | ≥ 96% | 49.70% |
